# Silent Pulmonary Artery Dissection in a patient with old Pulmonary Balloon Valvuloplasty

**Published:** 2014-09

**Authors:** Marzieh Nikparvar, Mozhgan Parsaee, Majid Maleki, Azin Alizadehasl, Rasoul Azarfarin

**Affiliations:** 1 Cardiovascular Research Center, Hormozgan University of Medical Sciences, Bandar Abbas, Iran;; 2 Shaheed Rajaie Cardiovascular Medical and Research Center, Department of Echocardiography, Tehran, Iran;; 3 Shaheed Rajaei Cardiovascular Medical and Research Center, Iran University of Medical Sciences, Tehran, Iran

**Keywords:** Dissection, Balloon valvuloplasty, Echocardiography

## Abstract

Percutaneous pulmonary balloon valvuloplasty (PBV) remains the treatment of choice for pulmonary stenosis (PS). This procedure is effective, safe and gives excellent results. Pulmonary artery (PA) dissection is a rare complication of PBV. This report is a case of an asymptomatic 17-year-old male with a history of PBV due to severe PS dating back to fifteen years ago. During recent echocardiography, an intimal flap was detected in the main PA and entry site was clearly seen by contrast study.

## Introduction

Almost all pulmonary valve stenisis (PS) have congenital source and its acquired form is extremely rare.^1^ Most patients with gradients of 50-79 mmHg will ultimately require treatment.^2^ Pulmonic stenosis is best treated with PBV which has a good long-term result,^3^ since the technique is relatively straight forward. Balloon diameter should be at least 10% to 20% longer than the pulmonic valve annulus.^3^ In contrast to the aortic value, pulmonic valve is elastic and often requires oversize to achieve adequate results.^3^ Maximum measure of balloon inflation should be a factor of 1.2 to 1.4 of the approximate size of the pulmonary annulus.^3^ The goal of the procedure is a final peak to peak valvular gradients less than 30 mmHg by cardiac catheterization.^4^ Echocardiography shows the results and recognizes rare complications of PBV such as PA dissection; but the use of nonstandard views may be necessary since diagnosis of PA dissection is difficult and it is classically recognized during postmortem examination.^5^

PA dissection is an extremely rare and fatal disease. PA dissection has most commonly been described in patients with structural heart disease such as congenital disorders or rheumatismal involvement; and in those with pulmonary thrombosis and chronic obstructive pulmonary disease. Catheter-induced PA dissection has been reported as extreme rare cause.^6^

## Case Report

This report is a case of a 17-year-old male with a history of PBV dating back to fifteen years ago. The patient had severe PS with a peak pressure gradient of 126 mmHg. PBV was carried out on the patient with multipurpose A2 balloon number 15. After the procedure, peak pressure gradient decreased to 37 mmHg. Annular size of pulmonic valve was 12 mm and standard echocardiographic evaluation showed mild residual valvular PS with mild pulmonary valve regurgitation.

**Figure 1:**
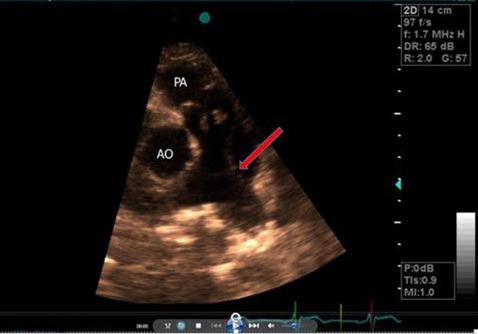
short axis parasternal view showing thickened and dome shaped pulmonary valve and intimal flap in lateral wall of main pulmonary artery (Red arrow).

**Figure 2:**
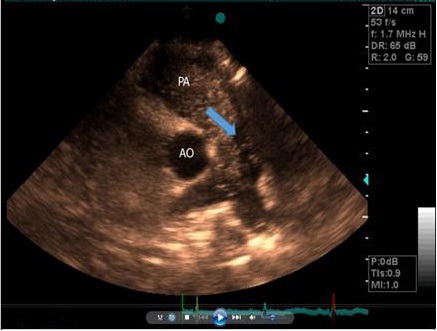
short axis parasternal view after agitated saline injection showing free space site in lateral of main PA in favor of false lumen with delay filling (Blue arrow).

**Figure 3:**
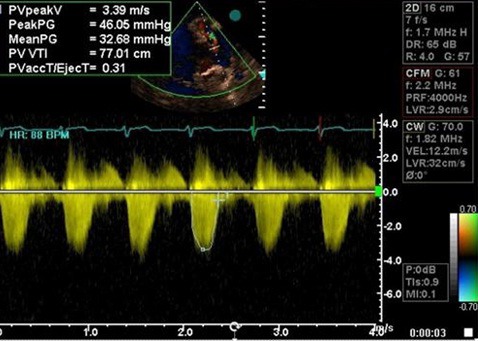
CW Doppler study across pulmonary valve showing mild to moderate valvular PS (PPG=46 mmHg).

The patient was asymptomatic and serial follow up echocardiography showed no other complications. At the recent presentation, fifteen years after valvuloplasty, the patient was still asymptomatic and had a systolic murmur on the upper left sternal border. The transthoracic echocardiography showed mild RV enlargement with normal function, mild to moderate residual PS (peak PG=46 mmHg, mean PG=32 mmHg), moderate PI, mild TR and RVSP~50 mmHg. An intimal detachment was seen in lateral side of PA trunk from ST junction to PA bifurcation (PA annular=2.4 cm); although this complication was revealed in off axis views and then in RVOT view. The entry site was seen clearly by contrast study (figures 1-3).

The study plan for managing such asymptomatic patient with only “mild RV dilatation, normal RV function and moderate pulmonary regurgitation” was conservative with periodic echocardiographic follow-up. PA dissection could be clearly distinguished by TTE without any ambiguity on the diagnosis. Considering the proposed treatment plan in conjunction with patient’s financial restrictions, TEE or other extra modalities (multi-detector computed tomography or magnetic resonance imaging) were not carried out.

## Discussion

Since the first introduction of pulmonary balloon valvuloplasty (PBV) by Kanetal in 1982, PBV has become the treatment of choice for pulmonary valvular stenosis in newborns and children.^7^ In majority of patients, the use of oversized balloons at least 10% to 20% larger than the pulmonic valve annulus is recommended.^3^

Major complications of PBV are rare as previously reported in infants and children.^8^ Such complications includes death (0.2%), cardiac perforation (0.1%) and tricuspid insufficiency (0.2%).^8^ Except for transient arrhythmias, no serious complications were encountered among the reported series with any early or late death.^8^ Furthermore, PA dissection is a rare complication of balloon valvuloplasty. It is often associated with structural heart disease (e.g. congenital, rheumatic heart disease, and/or pulmonary hypertension) as well as chest trauma.^9^ In 2005, Khattar et al. examined 63 patients with pulmonary artery dissection who were reported over the past two centuries. It was found that only 8 (13%) cases were diagnosed while alive and only 7 (11%) were diagnosed as having idiopathic or unspecified dissection.^6^ Echocardiography could be an excellent modality for evaluating the results and recognizing the complication of balloon valvuloplasty.

However using nonstandard views may be necessary, especially in patients with poor-views.

In the patient of this report, the intimal detachment was seen in off axis views and outflow view, but in parasternal short axis view it was seen with difficulty. This is the most probable reason for not describing the complication in the previous echocardiography reports. Clearly, experience of echocardiographer as well as equipment quality is critical for a better visual station of the pulmonary artery.

The optimal management of pulmonary artery dissection is surgery, since PA dissection is frequently presented as cardiogenic shock or sudden death.^10^ It is worth mentioning that these presentations have been frequently reported on the base of pulmonary artery hypertension or aneurysm. Catheter-induced PA dissection is extremely rare and consequently optimal management of it remains ambiguous. With respect to asymptomatic of the current patient without PA dilation or pulmonary artery hypertension, conservative medical therapy with close follow up was the preferred option. Interestingly, the patient was consistently asymptomatic two years after recognition of the problem without noticeable change of the main PA diameter.

Qingbao Li et al. reported a child with severe PS whom pulmonary balloon valvoplasty (PBV) was performed. Follow up transthoracic echocardiography after one month of the procedure revealed dilated PA with obvious flap inside the main PA. The patient was nominated for surgery despite the fact that the patient was asymptomatic as they found more dilation of the main PA than prior to the valvoplasty.^11^

It is believed that the natural course of PA dissection is not known due to the paucity of cases in the literature.

Conservative treatment with follow up echocardiography for stable pulmonary balloon valvoplasty is recommended. Surgery would be proposed for patients who show further dilation of the PA or occurrence of related symptoms.
